# Carpal Tunnel Syndrome Associated with Oral Bisphosphonates. A Population-Based Cohort Study

**DOI:** 10.1371/journal.pone.0146772

**Published:** 2016-01-14

**Authors:** Alfonso Carvajal, Luis H. Martín Arias, María Sáinz, Antonio Escudero, Inmaculada Fierro, Odile Sauzet, Victoria R. Cornelius, Mariam Molokhia

**Affiliations:** 1 Centro de Estudios sobre la Seguridad de los Medicamentos, Universidad de Valladolid, Valladolid, Spain; 2 AG Epidemiologie and International Public Health, Universität Bielefeld, Bielefeld, Germany; 3 Department of Primary Care & Public Health Sciences, King’s College London, London, United Kingdom; College of Medicine, National Taiwan University, TAIWAN

## Abstract

**Background:**

Bisphosphonates are widely used to prevent osteoporotic fractures. Some severe musculoskeletal reactions have been described with this medication; among them, some cases of carpal tunnel syndrome. Thus, the aim of this study was to explore whether bisphosphonates may be associated with this syndrome.

**Methods:**

A cohort study was conducted to compare exposed to unexposed women; the exposed group was that composed of women having received at least one prescription of an oral bisphosphonate. For the purpose, we used information from The Health Improvement Network (THIN) database. The outcome of interest was defined as those women diagnosed with carpal tunnel syndrome. A survival analysis was performed; the Cox proportional hazard model was used to calculate hazard ratios and 95% confidence intervals, and to adjust for identified confounding variables.

**Results:**

Out of a sample of 59,475 women older than 51 years, 19,825 were treated with bisphosphonates during the period studied. No differences in age distribution or mean follow-up time were observed between the two groups in comparison. Overall, there were 572 women diagnosed with carpal tunnel syndrome, 242 (1.2%) in the group exposed to bisphosphonates, and 330 (0.8%) in the unexposed. An adjusted hazard ratio of developing carpal tunnel syndrome of 1.38 (95%CI, 1.15–1.64) was found for women exposed to bisphosphonates; no significant changes in the hazard ratios were found when considering different levels of bisphosphonate exposure.

**Conclusions:**

An increased risk of carpal tunnel syndrome is associated with the use of bisphosphonates in postmenopausal women.

## Background

Bisphosphonates have proven to avoid osteoporotic fractures and are widely used to prevent these fractures particularly in postmenopausal women [1]. Prevalence of bisphosphonate use has considerably increased in the last years, due in part to demographic trends in older female population and to concerns of Hormone Replacement Therapy safety [2]. However, concerns have also been raised about serious adverse effects of this medication; these include osteonecrosis of the jaw, atrial fibrillation, atypical fractures [3] and, recently, conflicting evidence regarding risk of esophageal cancer [4]. Besides, a link between taking bisphosphonates and a raised risk of severe musculoskeletal pain has been established [5, 6]; indeed, prescribing information for all bisphosphonates already includes warnings of this particular risk [7, 8]. Additionally, cases of synovitis, a well-recognized cause of carpal tunnel syndrome (CTS) (acting through increased local pressure), have occasionally been reported [9–11].

CTS is the commonest peripheral nerve condition and has considerable employment and healthcare costs [12]; it is caused by elevated pressure in the carpal tunnel, and this in turn produces compression of the median nerve, resulting in impaired nerve conduction, paresthesia and pain [13]. The role of occupation has been the focus of much attention as a possible risk factor [14]. In a study carried out with a twin registry in the UK, a genetic predisposition was found to be the single strongest factor in predicting CTS [15]; BMI increase has also been consistently identified as a risk factor [16, 17]. Other medications such as lithium [18], oral contraceptives [19], exemestene [20]—an aromatase inhibitor used to treat breast cancer—and dabigatran [21] have occasionally been involved in CTS. The aim of this study was to explore whether bisphosphonates may be associated with carpal tunnel syndrome.

## Methods

### Design and setting

We conducted a retrospective cohort study by using data from The Health Improvement Network (THIN) database (www.thin-uk.com). This is a large database of anonymized computerized primary health care records of patients throughout the UK; it contains the electronic clinical records of more than 9.1 million patients (3.4 million of whom are alive). Anonymized patients’ data are collected from over 479 participating practices that are broadly representative of UK general practices in terms of patients’ age and sex, practice size, and geographical distribution [22]; moreover, consultation and prescription rates recorded in the THIN database have been shown to be comparable with published estimates [23]. Every prescription issued by the general practitioner, all consultations with the general practitioner, test results and diagnoses from primary and secondary care, referrals to outpatient clinics, hospital admissions, and deaths are coded by the general practitioner and entered into the database, as are basic demographic data and certain lifestyle data, such as smoking status and alcohol intake; recorded diagnoses and symptoms are classified using Read codes [24]. The THIN database has been validated for pharmacoepidemiological research [25].

### Study population

Our study population was based on women over 51 years registered for ≥ 1 year between 09/25/1994 and 07/07/2005 with records in the THIN database. Patients diagnosed with cancer or Paget’s disease, or registered for less than 1 year with their GP, were excluded.

### Exposure, index date and matching

In our cohort study we compared exposed to unexposed women; the exposed group was that composed of women having received at least one prescription of an oral bisphosphonate, i.e., they had a record of at least one prescription for any oral bisphosphonate preparation that is licensed in the UK for use in osteoporosis (British National Formulary section 6.6.2: sodium clodronate, disodium etidronate, and the aminobisphosphonates: alendronic acid, ibandronic acid and risedronate sodium); thus, IV bisphosphonates, such as zolendronic acid, whose main use was to treat bone metastases induced-fractures were not included. The date of the first prescription was the index date for exposed, and matched to the same date for the corresponding unexposed individuals; for every treated woman, two unexposed women, matched by age of treated woman at the index date, were taken as controls; exposed and unexposed women were also matched by practice, and any individual with previous history of carpal tunnel syndrome prior to bisphosphonate drug exposure was excluded from the analysis.

### Level of Exposure

Exposure was primarily defined as any exposure to bisphosphonates. Subsequently, the number of prescriptions issued was considered; it was stratified in 3 categories according to intensity of prescribing (number of prescriptions: 1–4, 5–16 and more than 16).

### Outcome

The outcome of interest was defined as those women diagnosed with CTS; women were defined as having CTS if, at any time during the observation period, they had a medical record of the syndrome (Read codes: F340.00—carpal tunnel syndrome; 7056400—endoscopic carpal tunnel release; 85BE.00—injection of carpal tunnel; 7056000—carpal tunnel release; 7056011—carpal tunnel decompression; 7056200 –re-release of carpal tunnel). The date of the first recorded code was considered the diagnosis date. A sensitivity analysis examined solely surgical related codes such as: endoscopic carpal tunnel release, carpal tunnel release, carpal tunnel decompression and re-release of carpal tunnel.

### Covariates

Based on the literature, covariates initially considered for adjusting in the analysis were: age, smoking status, alcohol intake, BMI, hypothyroidism, rheumatoid arthritis, all types of diabetes and number of GP visits over a defined time period. The information taken was that consigned closest to the index date; for the visits to the GP, the number was that within three months previous to the index date. Confounders were selected among those with a p<0.1 significance level in a previous univariable analysis. A forward stepwise method was applied to select confounders to be included in the final multivariable model.

### Statistical analysis

For survival analysis, the Cox proportional hazard model was used to calculate hazard ratios (HRs) and 95% CIs, and to adjust for identified confounding variables.

Risks were examined by category of bisphosphonate (amino- versus non aminobisphosphonate) and, where numbers permitted, by individual drug. Patients who were transferred out of the general practice or who died were censored, and the date of the censoring event was used as the end of follow-up time. To address missing values in the Cox regression analysis, multiple imputation method was performed; other for sensitivity analysis methods were also used, a complete-subject analysis, and the creation of a missing data category. We included interaction terms between exposure and each of the categorical variables considered; interaction terms between exposure and duration of follow-up was used to test the proportional hazard assumption, and the assumption was not shown to be violated (P>0.05 for all tests). To investigate dose response, separate analyses were conducted for the three categories established (1–4; 5–16 and > 16); each exposed group was compared with the corresponding matched unexposed group.

All analyses were conducted using SPSS software, version 19.0 (licenced for Universidad de Valladolid, 2013), and tests were performed at the 5% significance level.

## Results

Out of a sample of 59,475 women older than 51 years, 19,825 were treated with bisphosphonates during the period studied; this accounts for an exposed cohort of 51,245 person-years of follow-up. Distribution of prognostic factors between exposed and unexposed women after matching is presented in Table 1. No differences in age distribution or mean follow-up time were observed between the two groups in comparison. Overall, there were 572 women diagnosed with carpal tunnel syndrome, 242 (1.2%) in the group exposed to bisphosphonates, and 330 (0.8%) in the unexposed. An adjusted hazard ratio of developing carpal tunnel syndrome of 1.38 (95%CI, 1.15–1.64) was found for women exposed to bisphosphonates (Table 2); similar results were found when applying other methods to deal with missing values—complete subject analysis and missing data as a category. No significant changes in the hazard ratios were found when considering the levels of bisphosphonates exposure (Table 2). When considering those CTS codes referred to surgical procedures, the numbers of cases of CTS were 85 (0.4%) in the group exposed to bisphosphonates, and 115 (0.3%) in the unexposed; the crude and adjusted hazard ratios were, 1.49 (95%CI, 1.12–1.97) and 1.48 (95%CI, 1.09–2.00), respectively. Women exposed to aminobisphosphonates did not present a higher risk of CTS; adjusted hazard ratios of aminobisphosphonate-treated women (n = 15639) versus unexposed women and non-aminobisphosphonate-treated women (n = 2281) versus unexposed women were 1.36 (95%CI, 1.11–1.66) and 1.37 (95%CI, 0.95–1.96), respectively. For women exposed to alendronate (n = 11563), the most widely used bisphosphonate, the adjusted hazard ratio of CTS was 1.34 (95%CI, 1.08–1.65).

**Table 1:** Baseline characteristics. Distribution according to prognostic factors.

	Number (%)	
	Exposed (n = 19 825)	Unexposed (n = 39 650)
Age at Index Date (mean ± SD), years	72.1 ± 10.0	72.1 ± 10.0
<59	2647 (13.5)	5294 (13.4)
60–69	5134 (25.9)	10 269 (25.9)
70–89	11 402 (57.5)	22 800 (57.5)
≥90	642 (3.2)	1287 (3.3)
Follow-up (mean ± SD), years ^a^	2.6 ± 1.9	2.6 ± 1.9
Number of GP visits (mean ± SD)^b^	4.3 (3.8)	2.2 (2.9)
<2	4829 (24.4)	21 323 (53.8)
2–4	7582 (38.2)	12 368 (31.2)
≥5	7414 (37.4)	5959 (15.0)
Alcohol intake		
Non-heavy drinker	18 064 (91.1)	36 537 (92.1)
Heavy drinker	1728 (8.7)	3051 (7.7)
Missing^c^	33 (0.12)	62 (0.2)
BMI (kg/m^2^)		
<25.0	8974 (45.3)	13 117 (33.1)
25.0–29.9	5138 (25.9)	11 256 (28.4)
≥30	2317 (11.7)	6839 (17.2)
Missing	3396 (17.1)	8438 (21.3)
Smoking status		
Never	5865 (29.6)	11 429 (28.8)
Former	6027 (30.4)	11 734 (29.6)
Current	6387 (32.2)	11 928 (30.1)
Missing	1546 (7.8)	4559 (11.5)
Diabetes	3104 (15.7)	4774 (12.0)
Hypothyroidism	2139 (10.8)	3534 (8.9)
Rheumatoid Arthritis	1315 (6.6)	642 (1.6)

^a^ Time from Index Date to outcome or censorship

^b^ Visits within three months prior to Index Date

^c^ Heavy drinker recorded when the patient ever has a heavy drinker record (specific Read code or condition consistent with heavy alcohol consumption) and non-heavy drinker otherwise. When there was no consistent information to ascribe the case to one of the previous categories, data were considered as missing.

**Table 2:** Hazard of carpal tunnel syndrome associated with the intake of bisphosphonates.

	Exposed		Unexposed		HR (95% CI)	
	Cases	Person-years	Cases	Person-years	Crude ^a^	Adjusted ^b^^,^ ^c^
Any prescription	242	51 674	330	103 905	1.48 (1.25–1.75)	1.38 (1.15–1.64)
*Number of prescriptions*						
* 1–4*	*46*	*10 568*	*83*	*25 843*	*1*.*37 (0*.*96–1*.*97)*	*1*.*29 (0*.*88–1*.*89)*
* 5–16*	*73*	*14 860*	*98*	*31 134*	*1*.*54 (1*.*13–2*.*08)*	*1*.*34 (0*.*97–1*.*84)*
* ≥17*	*123*	*26 246*	*149*	*46 928*	*1*.*47 (1*.*16–1*.*87)*	*1*.*43 (1*.*11–1*.*85)*

^a^ Adjusted for age-group (52–59, 60–69, 70–89, ≥90)

^b^ Adjusted for BMI (<25, 25–29.9, ≥30), age-group (52–59, 60–69, 70–89, ≥90) and number of visits (<2, 2–4, ≥5)

^c^ -2 log likelihood, p<0.0001 for all models

A higher BMI was independently associated with a higher risk of CTS; adjusted hazard ratios for BMI 25.0–29.9 versus BMI<25 and BMI >30 versus BMI<25 were 1.28 (95%CI, 1.06–1.59) and 1.80 (95%CI, 1.46–2.23), respectively. No statistically significant relationship was found between diabetes, hypothyroidism or rheumatoid arthritis and risk of CTS after controlling for other risk factors.

## Discussion

In this cohort study with prospectively recorded information on prescribing of bisphosphonates, we found a statistically significant increased risk of CTS in women over 51 years of age treated with oral bisphosphonates. The risk was not related to the level of drug exposure, nor has it varied with the class of bisphosphonate used, whether amino- or non-aminobisphosphonate or even specific bisphosphonates such as alendronate. Our study was able to clearly confirm higher BMI as an independent risk factor for CTS; a higher BMI has been similarly identified in most of the observational studies upon CTS [16, 17]. However, though women with the highest BMIs are more likely to develop CTS, they are less prone to develop osteoporosis and subsequently less prone also to receive a bisphosphonate prescription; in fact, in our cohort, a lower percentage of women with the highest BMIs received bisphosphonate prescriptions (46% and 34% of women having BMI 25.0–29.9 and BMI >30 received bisphosphonate prescriptions, respectively, versus 68% for those having BMI <25). In our analysis comprising women whose median age was 72 years-old we did not observe diabetes, hypothyroidism and rheumatoid arthritis as significant risk factors for CTS; this is consistent with what has recently been observed in a large case-control study [26], aging appears to reduce the relative impact of the diseases commonly associated with CTS as the possible risk factors.

An alert highlighting the possibility of severe and sometimes incapacitating bone, joint, and/or musculoskeletal pain has been issued by the FDA [27]; indeed, prescription information for all bisphosphonates includes warnings of this particular risk [7, 8]. This severe musculoskeletal pain may occur within days, months or years after starting bisphosphonates. A series of 117 cases of severe musculoskeletal pain developing in adults on bisphosphonates has been published [5]. Moreover, cases of synovitis confirmed by positive re-challenge have also been reported [9–11] and may occur at any point after starting bisphosphonate therapy, similarly to the occurrence of the cases of CTS we identified.

Arthritis—one of the leading causes of CTS [10]—and its correlate arthralgia is one of the most reported adverse effects for bisphosphonates. Two facts would be able to explain biological plausibility of bisphosphonate-induced arthritis and synovitis. First, the appearance of inflammatory cytokines, in particular TNFα and IL6, has been observed after bisphosphonate treatment *in vitro* and in patients [28–31]; thus, for synovitis to occur, synovial macrophages would release those cytokines when bisphosphonates are present; the way this process is triggered remains unclear. Second, the skeletal half-life of various bisphosphonates is sometimes more than 10 years [32]; once bisphosphonates are buried in the skeleton, they will be released only when the bone is destroyed in the course of turnover. Being an inflammatory process which can be triggered by these substances in any moment during long periods would explain the fact that this reaction is not related to time or dose.

The strengths of the present study are its large sample size, the long period of follow-up, and the use of recorded prescription data rather than self-reported drug use, which may misclassify exposure.

Since bisphosphonates cannot be obtained without prescription in the United Kingdom, underestimation of its usage would seem unlikely; however, a limitation of the study is that drug adherence is unknown. In fact, since exposure does rely on prescriptions issued, overestimation of usage is possible, as compliance with bisphosphonate prescribing is known to be suboptimal [33]); if so, the real CTS risk associated with bisphosphonates would be higher. Additionally, an interval between prescription and intake might exist; however this information is unknown for the two compared groups. Also, we relied on relevant diagnostic codes from patients’ clinical files and CTS is a specialist diagnosis. However, as codes have a certain degree of unreliability, some misclassification might be inevitable; therefore, we considered those codes involving CTS surgical procedures which further supported the accuracy of the diagnoses—most surgical procedures would also require electrophysiological testing, although this is not routinely recorded in the notes—, finding no significant changes in our previous estimates. Furthermore, the treatment of missing data for BMI, the only important variable with a considerable number of missing data, was handled using the current recommended approaches to avoid the biases that occur when omitting individuals with incomplete data. We were not able to adjust for vocational activities as data was incomplete similarly data for chronic kidney disease was sparsely recorded; although we have adjusted for the number of visits, it is still possible, but unlikely, as a surveillance bias.

In summary, we have identified an increased risk of CTS associated with bisphosphonates; it is a novel finding with potential clinical impact. As increasing numbers of individuals, particularly postmenopausal women, are exposed to this medication, a large number of cases of this invalidating and costly condition might appear in excess. If confirmed, doctors should be aware to avoid prescribing this medication in those women with other risk factors for CTS such as elevated BMI or family history of the disease; regulatory authorities, in turn, should consider appropriate further safety advice regarding CTS risks with bisphosphonates.
